# Application of the ICP-OES and SEM-EDS Techniques for Elemental Analysis of Various Types of Cosmetic Products with Antiperspirant and Deodorant Properties Available on the EU Market

**DOI:** 10.3390/molecules30204050

**Published:** 2025-10-11

**Authors:** Elżbieta Maćkiewicz, Aleksandra Zimon, Aleksandra Pawlaczyk, Jadwiga Albińska, Małgorzata Iwona Szynkowska-Jóźwik

**Affiliations:** Institute of General and Ecological Chemistry, Faculty of Chemistry, Lodz University of Technology, Zeromskiego 114, 90-543 Lodz, Poland; aleksandra.zimon@p.lodz.pl (A.Z.); aleksandra.pawlaczyk@p.lodz.pl (A.P.); jadwiga.albinska@p.lodz.pl (J.A.); malgorzata.szynkowska@p.lodz.pl (M.I.S.-J.)

**Keywords:** deodorants, antiperspirants, elemental analysis, ICP-OES, SEM-EDS, aluminium, zirconium

## Abstract

Nowadays deodorants and antiperspirants play an important role in maintaining daily hygiene, exerting a substantial influence on both physical comfort and social functioning. Consequently, they can be regarded as a pivotal component of contemporary personal hygiene programs. The aim of this study was to undertake a comparative analysis of the elemental composition of diverse samples (72) of various roll-on deodorants and antiperspirants, sticks, and solid natural potassium–aluminium alums. These analyses were performed using ICP-OES and SEM-EDS techniques. The obtained results were then subjected to statistical and chemometric analysis. Studies demonstrated that Al and Zr were the most significant elements in the tested samples. Aluminium, a prevalent component in antiperspirants, was quantified in concentrations ranging from 0.9% to 4.4%, and in potassium–aluminium alums up to 4.7%. Aluminium and zirconium compounds were found to be the predominant elements in stick antiperspirants, with zirconium levels reaching up to 3%. The presence of lead was quantified in 35 of the 72 samples, with 19 samples exhibiting concentrations exceeding 1 mg/L. The highest lead level, reaching 15.90 mg/L, was found in potassium–aluminium alum. Furthermore, SEM-EDS analysis was conducted to verify the elemental composition, to provide data on additional ingredients, and to partially verify the information contained on the product labels.

## 1. Introduction

Deodorants and antiperspirants represent a highly diverse group of cosmetic products that have gained significant popularity in developed countries. Deodorants and antiperspirants are two distinct types of cosmetic products that, despite being frequently confused, serve different functions in daily hygiene [1]. Deodorants are formulated to mask or neutralise the malodorous nature of perspiration, chiefly by virtue of the antibacterial properties inherent within their composition. Sweat itself is devoid of any odour; however, upon contact with bacteria residing on the skin—particularly in the armpit area—volatile compounds are formed, which are responsible for the unpleasant odour [1,2,3,4,5]. Antiperspirants, conversely, are formulated with compounds containing aluminium or aluminium and zirconium, which serve to create a transient barrier within the ducts of the sweat glands, thereby reducing sweat secretion [1,6,7]. This phenomenon represents a temporary effect, the nature of which dissipates either as the stratum corneum of the epidermis undergoes desquamation or in cases of profuse perspiring. The fundamental distinction between these two products lies in their respective mechanisms of action. Deodorants impede bacterial proliferation, thereby mitigating malodorous emanations. Antiperspirants, in contrast, are designed to regulate perspiration. The first modern deodorant, comprising waxes and zinc oxide as active ingredients, was marketed in 1888 in the United States under the brand name “Mum” and was applied using a cotton ball [1,4]. The advent of the roll-on applicator in the 1940s marked a pivotal innovation, significantly enhancing the convenience and popularity of antiperspirant utilisation. Despite their differences in effectiveness, deodorants and antiperspirants are both essential elements of modern daily hygiene and comfort.

Deodorants and antiperspirants are available in a variety of forms, including aerosol, roll-on, stick, pump, gel and cream [1]. Recently, mineral deodorants containing aluminium–potassium alum have become increasingly popular [6,8]. The composition of deodorants and antiperspirants differs significantly in most cases. Deodorants are principally composed of substances that exhibit bacteriostatic properties, including triclosan, alcohols (aliphatic and glycols), the aforementioned alums, metal-containing compounds and fragrances that serve to neutralise unpleasant odours [9]. Antiperspirants, by contrast, contain aluminium compounds such as aluminium chloride, aluminium chlorohydrate, aluminium sesquichlorohydrate, or aluminium lactate, as well as aluminium–zirconium compounds. The latter occur in the form of common hydroxychlorides, such as aluminium zirconium pentachlorhydrate, or most commonly, in the form of a complex with glycine, such as aluminium zirconium tetrachlorohydrex Gly, aluminium zirconium octachlorohydrex Gly [1,4]. It is also noteworthy that the composition of deodorants and antiperspirants is primarily determined by their formulation. For instance, aluminium–zirconium compounds are prohibited in antiperspirants in the form of a spray due to the possibility of inhalation [6,10].

According to Regulation (EC) No. 1223/2009 of the European Parliament and the Council on Cosmetic Products (Annex III), the use of aluminium and zirconium compounds in the form of chloride, hydroxide complexes and aluminium zirconium chloride hydroxide glycine complexes is permitted in antiperspirants, provided that the amounts do not exceed 20% of the compound as anhydrous aluminium zirconium chloride hydroxide and 5.4% as zirconium, whereby the ratio of the number of aluminium atoms to that of zirconium atoms must be between 2 and 10 and the ratio of the number of (Al + Zr) atoms to that of chlorine atoms must be between 0.9 and 2.1 [10]. In addition, in 2019, the Scientific Committee on Consumer Safety (SCCS) published an opinion on the safety of aluminium use in cosmetic antiperspirant products. In view of the new data, the SCCS concluded that aluminium compounds are safe in antiperspirants, and the use of aluminium compounds is safe at the following equivalent aluminium concentrations of up to 6.25% in non-spray deodorants or non-spray antiperspirants and 10.60% in spray deodorants or spray antiperspirants [11].

A considerable degree of uncertainty surrounds the use of antiperspirants. Aluminium’s free metal cation, Al^3+^ (aq), is highly biologically reactive and biologically available. In contrast, biologically available aluminium is non-essential and essentially toxic. Biologically reactive aluminium is present throughout the human body. While its acute toxicity is rare, much less is known about chronic aluminium intoxication [12]. The most recent research findings indicate that the extensive utilisation of aluminium, encompassing its presence in cosmetic products, could constitute a primary pathway through which the human body is exposed to this element. A number of scientific studies have indicated that aluminium, an elemental constituent of antiperspirants, may potentially induce breast cancer, among other health complications [1,13,14,15,16,17]. Further research is required to address the existing knowledge gaps and provide a more definitive understanding of the potential association between antiperspirant use and the risk of breast cancer. In contrast, alternative views posit that the degree of aluminium penetration through the skin is minimal, and that the moderate use of antiperspirants on non-irritated skin will not result in any adverse health effects [11,18].

In recent years, alum has seen a surge in popularity, particularly potassium alum, among individuals seeking natural and safe alternatives to conventional antiperspirants [6,8,19]. This trend is particularly noticeable among those adhering to a vegan lifestyle and those who are mindful of their environmental impact, opting for eco-friendly cosmetics that are free from certain ingredients, such as plastic, parabens, aluminium hydrochloride, and alcohol [5]. Alum has a long history, with its use dating back to ancient Egypt and Rome. In these historical periods, alum was employed in various applications, including the treatment of wounds, the purification of water, and as a preservative. In the contemporary era, there has been a resurgence of interest in these products, with a re-emergence as a minimalist, effective, and natural alternative to synthetic cosmetics. Alums are natural mineral salts that have been utilised for millennia in diverse fields, ranging from folk medicine and tanning to cosmetics. It is evident that both substances possess a comparable chemical structure, with the primary distinction lying in the presence of potassium within their respective compositions. Potassium alum (KAl(SO_4_)_2_·12H_2_O) is more commonly used in cosmetics and is available in the form of natural crystals [19]. When moistened with water, these crystals serve as a simple, odorless deodorant. Alum’s mechanisms of action are based on several factors. The substance exhibits strong astringent properties, meaning it has the effect of gently shrinking skin pores, thereby reducing excessive sweating without completely blocking it. This is in contrast to traditional antiperspirants that primarily contain aluminium hydrochloride. Another salient aspect pertains to its antibacterial properties, wherein alum has been demonstrated to effectively impede the proliferation of bacteria responsible for the malodorous nature of perspiration. Notably, the product does not mask odours, but rather acts to prevent them. The substance is devoid of alcohol and fragrance, does not cause irritation, and leaves no marks on clothing. In recent years, alum has gained immense popularity among those opting for natural and conscious skincare. As a mineral-based product, it is fully vegan and is often offered unpackaged or in reusable packaging, thus aligning perfectly with the zero-waste and clean beauty philosophy [5,19]. Alum crystal has been demonstrated to have a service life of up to several months, thus making it both an environmentally sound and economical solution. A growing number of companies, encompassing both small manufacturers and large eco-friendly brands, are incorporating alum into their products, frequently accompanied by certificates attesting to its naturalness and ethical origins. Nevertheless, it is imperative to emphasize that the nomenclature employed for alum products (potassium alum as opposed to potassium–aluminium alum) is potentially misleading. Additionally, they are marketed as aluminium-free deodorants, despite the fact that they do not contain any aluminium. However, it is imperative for users to acknowledge the relatively high aluminium content inherent in these products [5,8].

A number of studies have been conducted that concentrate on the monitoring of aluminium and zirconium in antiperspirants that are available on local markets. Furthermore, research has been undertaken into the presence of other toxic metals, including cadmium and lead. These analyses utilise dedicated elemental analysis methods, such as ICP-OES [20,21], AAS [22,23], colorimetry methods [24], a spot test together with diffuse reflectance spectroscopy [25], or electroanalytical methods [26]. It is important to note, however, that the testing was conducted on a limited number of samples in a single form of occurrence, namely spray or roll-on, and, equally significantly, the focus was predominantly on aluminium, with zirconium being largely excluded.

The sensitive and selective electrochemical flow injection analysis (FIA) technique was used to analyse the concentration of aluminium hydrochloride (AHC) present in antiperspirants [26]. Four different samples of real antiperspirants, with and without ACH, were tested, and the obtained values were found to be comparable to those listed on the product labels, demonstrating good agreement between them. Da Costa et al. developed a novel procedure for the direct analysis of nine liquid deodorant samples, determining the elements Al, As, Ba, Cd, Cu, Fe, Mg, Mn, Ni, Pb, Sc, Ti, V, Zn, and Zr using ICP-OES. This method features a rapid, straightforward, and reproducible sample preparation step that does not necessitate any specialised equipment [20]. This method is also less prone to contamination or loss of analytes. The aluminium contents obtained in the tested deodorants ranged from 0.010 to 7.0 mg/kg, while for Zr these values ranged from <0.04 to 0.500 mg/kg. For heavy metals such as Cd and Pb, no values higher than the LOQ were observed. The values obtained for Cd and Pb were both found to be less than 0.001 and less than 0.76, respectively [20]. In the field of analytical chemistry, other researchers have developed a novel method for the quantification of aluminium hydrochloride (AHC) in antiperspirants [25]. This method is notable for its low cost, portability and environmental friendliness. It involves a spot test on a paper platform, in conjunction with diffuse reflectance spectroscopy. The method has been validated, and its reliability has been demonstrated. The concentrations of aluminium hydrochloride obtained during the measurements did not exceed the national permissible limits of 25% (in Brazil) [25]. Teixeira et al. examined eight roll-on antiperspirant samples employing both the proposed colorimetric method and the established comparative method of flame atomic absorption spectrometry (FAAS) [24]. An analytical environmental assessment was also performed for both methods, and the developed procedure was found to be the more environmentally friendly approach due to miniaturization, low sample consumption, minimal waste, and high sample throughput. The obtained results did not differ statistically at the 95% confidence level. The obtained Al(III) concentration ranges from 10.82 to 36.61 g/L [24].

A review of the existing scientific literature indicates a paucity of data concerning the testing of a larger number of antiperspirant and deodorant samples that are more representative of the general population. In addition, there is a need for a broader analysis of the heavy metals and other ingredients contained within these products, as well as statistical and chemometric analysis. The aim of the present study was to assess the differences in the elemental composition of cosmetic products available on the European market depending on the type of product, the form in which it occurs and the type of active substance, with a particular emphasis on the concentrations of aluminium (Al) and zirconium (Zr).

## 2. Results and Discussion

### 2.1. General Characteristics of the Elemental Composition of Cosmetic Products with Antiperspirant and Deodorant Properties

A total of 23 elements were examined in the analysis, however, as cadmium was not detected in any of the samples, the results for this element were not quantified and were consequently excluded from the subsequent analysis. The results obtained demonstrate the general elemental composition of cosmetic products with deodorising and antiperspirant properties. The analysis enabled the characterisation of their overall elemental constituents, contributing to a more comprehensive evaluation of their composition. To ensure clarity and reproducibility of the study, Section 3 (Materials and Methods) with Section 3.1 (Samples) includes a complete compilation of the investigated samples, together with their systematic classification. The subdivision criteria and rationale are also described in this section.

The maximum value of 4.682% for Al was obtained for a solid potassium aluminium alum deodorant. Furthermore, more than 42% of the samples had aluminium (Al) concentrations above 3%, while only slightly more than 5% (four samples) had concentrations below 0.1%. Three of these four samples contained no aluminium according to the product label. It was a sample of roll-on deodorant for men. The only active deodorant ingredient was alcohol, and no aluminium-containing chemicals were found in the declared composition.

The highest zirconium (Zr) concentration obtained was 3%, which was found in a sample of women’s antiperspirant stick containing aluminium–zirconium tetrachlorohydrex GLY as the antiperspirant ingredient. It should also be noted that only 11% of the tested samples contained more than 0.5% zirconium. All of these samples contained aluminium–zirconium compounds in their composition. However, elevated Zr content was found in some samples despite the product label not listing any chemical compounds containing this element. For example, two men’s antiperspirant samples, one in the stick form and one in the roll-on form, had Zr concentrations of 0.357% and 0.4541%, respectively.

In the following sections of this publication a more detailed analysis of the Al and Zr concentrations in the tested samples will be presented.

The highest silver (Ag) concentration of 218.4 mg/L was obtained for an antiperspirant stick product containing an aluminium–zirconium compound as the active ingredient. However, no silver-containing chemicals were found in the labelled composition. Five other samples also had high concentrations of Ag (100–200 mg/L). Like the first sample with the highest Ag concentration, all of these samples were in a stick formulation with an aluminium–zirconium compound as the antiperspirant active ingredient. Silver, most often in the form of nanoparticles, is currently widely used in many areas of life and industry due to its antibacterial and antifungal properties. These properties are utilised in biotechnology, bioengineering and textile engineering [27,28,29]. Furthermore, nanosilver is used in cosmetics as an ingredient to enhance their healing, bactericidal, and fungicidal properties.

A variety of concentrations was obtained for the typical macroelements that are present in nature in high concentrations, including calcium (Ca), magnesium (Mg), potassium (K), phosphorus (P), and sulphur (S). Calcium and magnesium were present in low concentrations in the tested samples. The median value of Ca concentration was found to be 18.78 mg/L, while the median value of Mg concentration was found to be significantly lower, at 0.022 mg/L. However, elevated concentrations of calcium and magnesium were only detected in individual samples, likely attributable to the presence of naturally or biomimetic occurring chemical compounds. The maximum calcium content of 5692 mg/L was recorded for the sample which contains calcium/magnesium/zinc hydroxyapatite, oryza sativa (rice) starch, diatomaceous earth, clay minerals, and calcium chloride. Hydroxyapatite, a biomimetic material, is composed of calcium, magnesium, and zinc. While its origin is synthetic, it mimics the natural origin. A novel deodorising material has been developed, based on modified Ca_10_(PO_4_)_6_(OH)_2_ hydroxyapatite structures (HA), which are frequently combined with zinc PCA (Zinc Pyrrolidone Carboxylic Acid) in novel deodorants, acting synergistically, exerting astringent, deodorising, and bacteriostatic effects [30]. This is a women’s deodorant that is distinguished from other products by virtue of its markedly divergent composition, with the manufacturer asserting that the majority of its ingredients are of natural origin. The highest recorded value for Mg, 1111 mg/L, was obtained for the women’s antiperspirant stick. The manufacturer of this product asserts the presence of talc in its composition, a chemical compound that is also referred to as magnesium hydroxysilicate. The situation with potassium is more complex. On the one hand, it is a macroelement that occurs commonly in high concentrations. On the other hand, among the samples there are potassium alums that contain high concentrations of K. The maximum potassium content in the tested samples was 7.110%; however, this sample is among the group of samples with high K concentrations, above 5% (nine samples in total). The analysis revealed that seven of the samples were aluminium–potassium alums in the solid form, while the remaining two samples were roll-on products. It was observed that alum was the second most significant ingredient in two of the roll-on products, both of which were from the same company. Among the samples devoid of alum, the maximum K concentration was obtained for a universal sweat blocker that, as per the product label, contains potassium sorbate. As a popular preservative in food and cosmetic products, E202 is a common ingredient in many products [31]. A comparable scenario is observed in sulphur, as it is an integral component of alums. The maximum concentration achieved for sulphur is significantly higher than that of K, reaching 11.28%. For sulphur, concentrations in excess of 8% were obtained for nine samples. In a manner analogous to K, these samples were potassium–aluminium alums in the solid (mineral) or roll-on form. Conversely, the concentrations of phosphorus were predominantly minimal, with a median value of 2.260 mg/L. The only cosmetic product for which a higher P concentration was found than the others (3230 mg/L) is the previously described sample of a roll-on deodorant, where the main ingredient is the compound hydroxyapatite Ca, Mg and Zn. The samples that were analysed contained trace amounts of various elements, including Co, Cr, Cu, Fe, Mn, Ni, and Zn. For cobalt, the majority of results were below the limit of quantification (LOQ), with a maximum value of 0.569 mg/L observed for a roll-on antiperspirant sample. With regard to the presence of chromium, the majority of samples exhibited trace quantities of this element, with concentrations ranging below 2 mg/L. The maximum value for Cr of 5.561 mg/L was obtained for a solid sample of potassium aluminium alum. For one sample, the aforementioned universal sweat blocker, a maximum Cu value of 45 mg/L was obtained. For the remaining samples, these values were largely below 1 mg/L or below the limit of detection. For iron, the median value was 13.33 mg/L, with the maximum value obtained for a roll-on deodorant sample containing aluminium–potassium alum as the active ingredient and argania spinosa kernel oil, which is rich in iron [32]. With regard to the manganese content of the samples that were analysed, the median value was found to be 0.335 mg/L, while the maximum value obtained was 2.537 mg/L for the sample containing calcium/magnesium/zinc hydroxyapatite. For nickel, which plays a small but important role as a microelement and at the same time can be a toxic and strongly allergenic element, a median value of 0.212 mg/L was obtained in the tested population of samples, with one of the obtained values being significantly higher and amounting to 12.26 mg/L. This was a sample of a roll-on antiperspirant for men. As previously stated, zinc is an element that was utilised as a component in the inaugural cosmetic product with deodorant properties. Among the samples that were analysed, only one was characterised by a high zinc concentration of 1854 mg/L. This was the previously reported sample containing calcium/magnesium/zinc hydroxyapatite and zinc PCA, which have deodorising and antibacterial properties, as previously described in this publication [31]. The analysis of the results for other elements, including Ba, Sb, Sn, Sr, and Ti, revealed that the majority of concentrations were found to be close to the detection limit or slightly higher. According to EU regulations, the presence of Ba, Sn, Sr and Ti compounds in cosmetic products is permitted, with specific exceptions, while antimony and its compounds are prohibited [10]. The highest and only such result of Sb concentration in the tested samples was 3.700 mg/L, and this refers to a roll-on antiperspirant sample with aluminium–zirconium compounds as the active form. The final issue pertains to the presence of typical heavy metals such as Cd and Pb. As previously stated, no concentrations greater than the LOQ were obtained for cadmium; however, a significantly high value of 15.90 mg/L was observed for Pb. For 35 of the 72 samples, i.e., almost 50%, the value was above the LOQ, and for 19, i.e., almost 30%, the value was above 1 mg/L. The maximum value was obtained for one of the samples of solid potassium aluminium alum. It is also noteworthy that the regulation stipulates the absence of lead in cosmetic products [10].

### 2.2. Statistical Analysis

#### 2.2.1. The Classification According to the Form of Cosmetic Products

**Figure 1:**
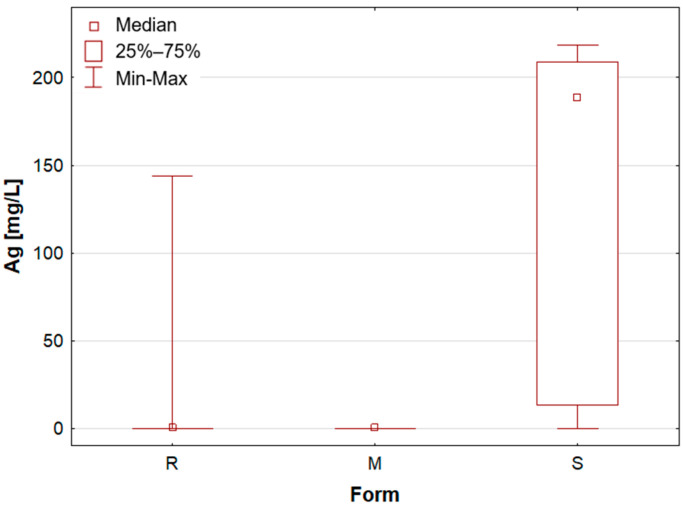
Box-and-whisker plot for Ag by form: R—roll-on; M—mineral; S—stick, mg/L.

**Figure 2:**
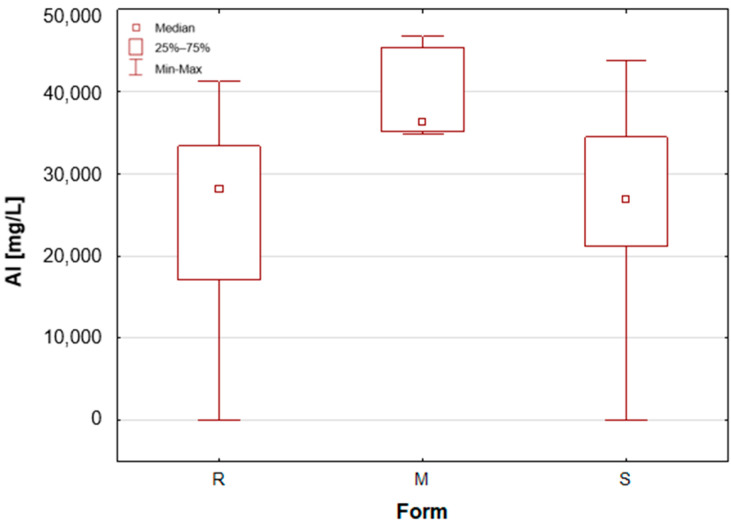
Box-and-whisker plot for Al by form: R—roll-on; M—mineral; S—stick, mg/L.

**Figure 3:**
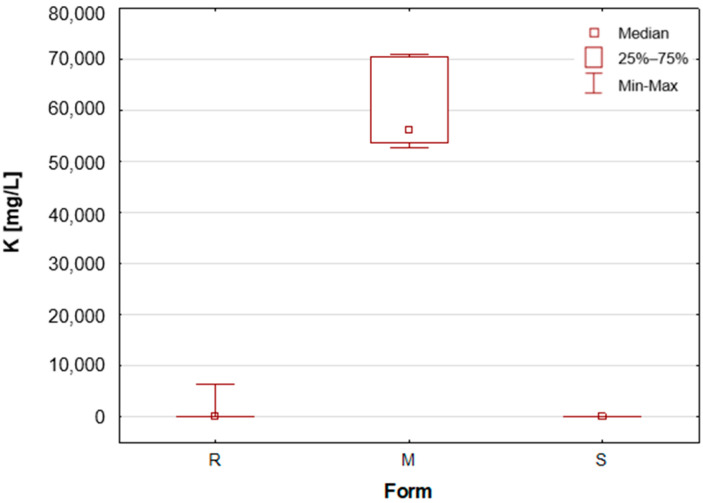
Box-and-whisker plot for K by form: R—roll-on; M—mineral; S—stick, mg/L.

**Figure 4:**
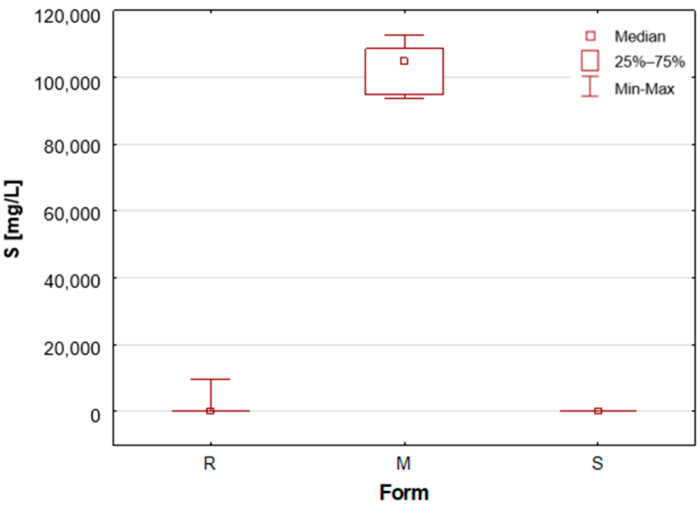
Box-and-whisker plot for S by form: R—roll-on; M—mineral; S—stick, mg/L.

**Figure 5:**
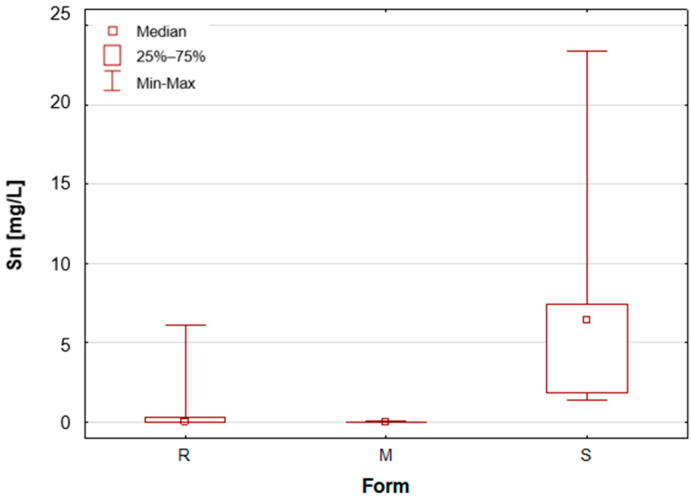
Box-and-whisker plot for Sn by form: R—roll-on; M—mineral; S—stick, mg/L.

**Figure 6:**
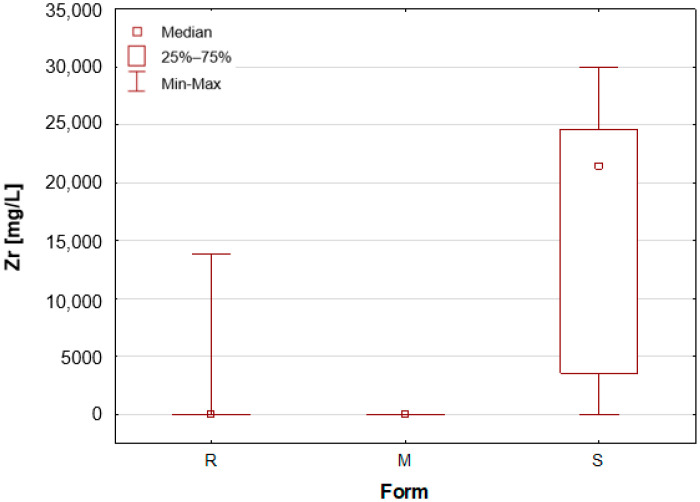
Box-and-whisker plot for Zr by form: R—roll-on; M—mineral; S—stick, mg/L.

As previously mentioned, the samples were categorised according to their form as roll-on—R, solid mineral (aluminium potassium alum)—M, and stick—S. A number of statistically significant differences were observed between the analysed groups. Table 1 presents the mean concentration and median value of each element content in deodorants and antiperspirants. Deodorants and antiperspirants are most commonly found in two forms: spray and roll-on. In the analysed sample group, the latter were the most numerous, with 58 samples. These products characteristically possess a creamy consistency, with instances of a watery consistency being less prevalent. The remaining two groups, i.e., mineral and stick, are represented by only seven samples. In addition, box-and-whisker plots are presented for chosen elements (Figure 1, Figure 2, Figure 3, Figure 4, Figure 5 and Figure 6).

Upon the division of the tested deodorant and antiperspirant samples according to their form of occurrence, a number of statistically significant differences were identified, namely for 11 out of 21 tested elements. For silver, only stick antiperspirants (S) exhibited a median value of 188.4 mg/L, the highest of all categories (Figure 1). For the other two groups, the median value was below the limit of quantification (LOQ), although several roll-on (R) samples also exhibited higher silver concentrations. These antiperspirant samples contained an aluminium and zirconium compound as the active ingredient. As previously mentioned, silver has antibacterial properties and is used as an additive in cosmetics and clothing. It is noteworthy that the median concentration of aluminium is observed to be at its highest in the samples categorised as aluminium–potassium alums in the solid form (M, Figure 2). The median value for this group is 3.628%, while a lower value of 2.820% is found in samples belonging to the roll-on group (R). The lowest value is found in those classified as sticks (S) at 2.684% (Table 1). The lowest aluminium (Al) content in the sticks is attributable to the high content of zirconium, as only two of the seven stick samples did not contain an aluminium–zirconium compound as an antiperspirant ingredient. The results obtained in this study are consistent with the extant literature data. The aluminium concentration in typical roll-on deodorants that do not contain aluminium is low, whereas this concentration increases significantly in antiperspirant products based on aluminium or aluminium–zirconium compounds. Literature data indicates that the aluminium concentration in roll-on antiperspirants can reach up to 3.7% [24]. The results obtained in this analysis suggest that these concentrations may be even higher, exceeding 4%. The group of solid potassium aluminium alums (M) is characterised by the highest potassium concentrations. The median K concentrations for this group was as high as 5.622%, while for the other two groups, these values were significantly lower, amounting to only about 40–50 mg/L (Table 1, Figure 3). The outcomes pertaining to sulphur concentrations are highly analogous. With regard to alums (M), the S median value was 10.47%, while for the other two groups, these values are found to be negligible, as illustrated in Table 1 and Figure 4. As with silver, tin exhibits a correlation with compounds present in samples containing aluminium–zirconium complexes, given that this group is distinguished by considerably higher concentrations of the element (Table 1, Figure 5). The median value of Sn concentrations for this group was 6.424 mg/L, with the highest value obtained in the analysis being as much as 23.395 mg/L. In contrast, zirconium has been found to be present primarily in stick antiperspirants. A statistical analysis reveals that for the stick group (S), the median Zr concentration is 2.142%, while for the other two groups, this value is found to be insignificantly low (Table 1, Figure 6). A study was conducted on three roll-on samples (R), and it was found that only these samples exhibited higher Zr concentrations (above 0.5%). The content of this element in the samples was declared on the product label.

#### 2.2.2. Classification by Type of Active Substance in the Tested Deodorants/Antiperspirants

**Figure 7:**
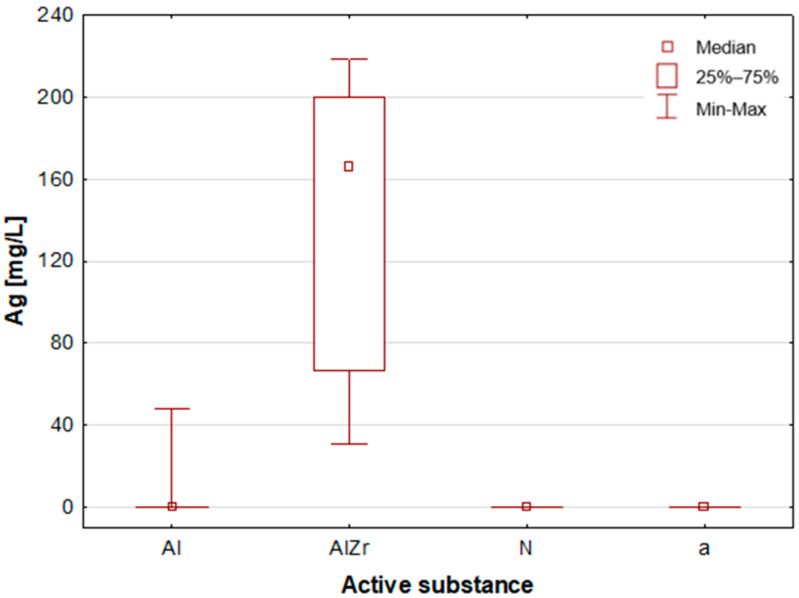
Box-and-whisker plot for Ag by type of primary deodorant or antiperspirant ingredient they contained: aluminium-based products (Al), aluminium–zirconium-based products (AlZr), natural compound-based products (N), alcohol-based products (a).

**Figure 8:**
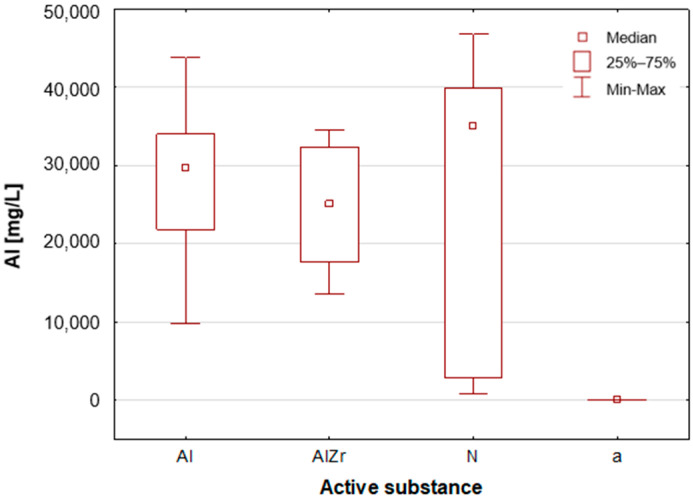
Box-and-whisker plot for Al by type of primary deodorant or antiperspirant ingredient they contained: aluminium-based products (Al), aluminium–zirconium-based products (AlZr), natural compound-based products (N), alcohol-based products (a).

**Figure 9:**
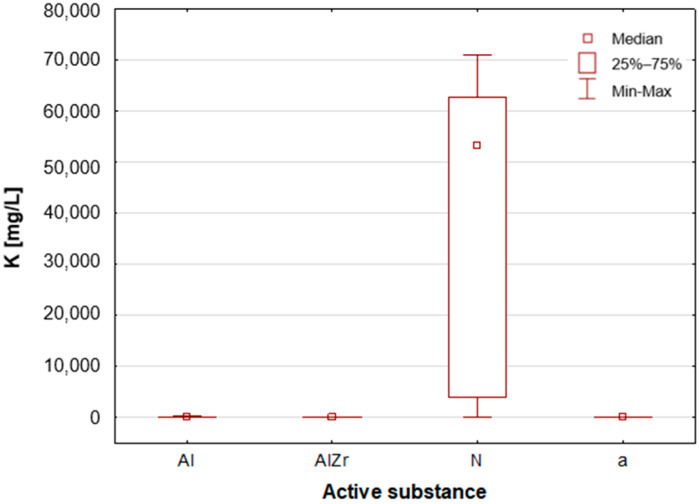
Box-and-whisker plot for K by type of primary deodorant or antiperspirant ingredient they contained: aluminium-based products (Al), aluminium–zirconium-based products (AlZr), natural compound-based products (N), alcohol-based products (a).

**Figure 10:**
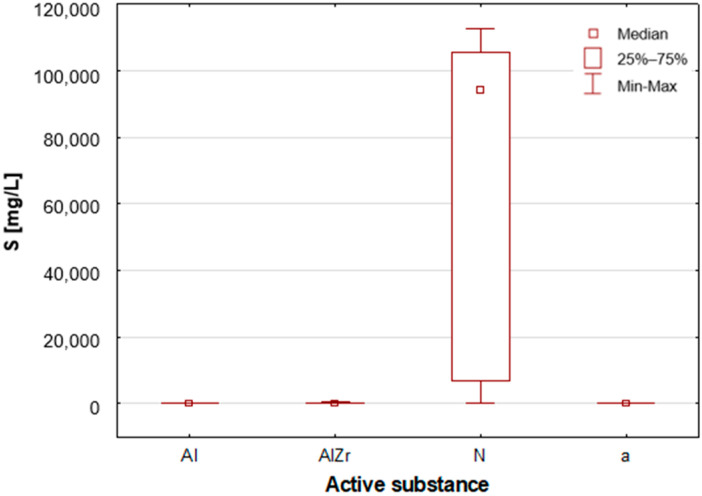
Box-and-whisker plot for S by type of primary deodorant or antiperspirant ingredient they contained: aluminium-based products (Al), aluminium–zirconium-based products (AlZr), natural compound-based products (N), alcohol-based products (a).

**Figure 11:**
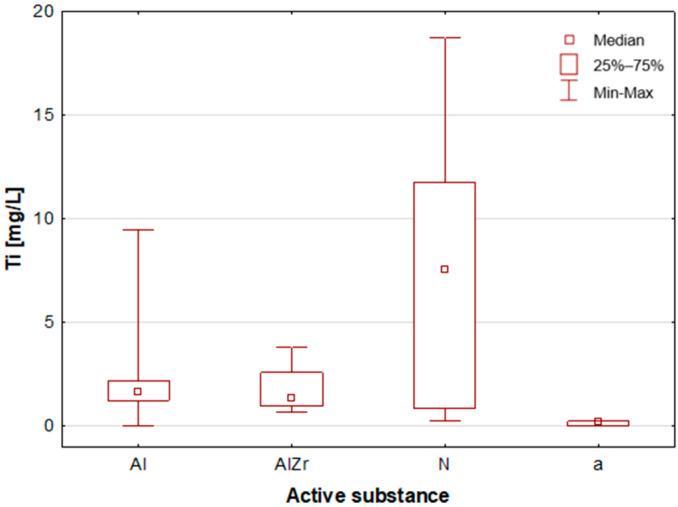
Box-and-whisker plot for Ti by type of primary deodorant or antiperspirant ingredient they contained: aluminium-based products (Al), aluminium–zirconium-based products (AlZr), natural compound-based products (N), alcohol-based products (a).

**Figure 12:**
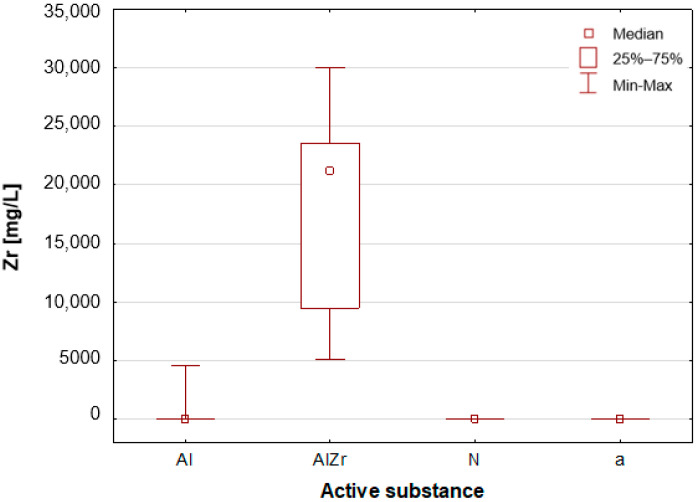
Box-and-whisker plot for Zr by type of primary deodorant or antiperspirant ingredient they contained: aluminium-based products (Al), aluminium–zirconium-based products (AlZr), natural compound-based products (N), alcohol-based products (a).

As previously stated, the samples were categorised according to the type of primary deodorant or antiperspirant ingredient they contained: aluminium-based products (Al), aluminium–zirconium-based products (AlZr), natural compound-based products (N), and alcohol-based products (a). A number of statistically significant differences were identified between the groups analysed. As illustrated in Table 2, the mean and median concentrations of each element in the deodorants and antiperspirants are presented. Figure 7, Figure 8, Figure 9, Figure 10, Figure 11 and Figure 12 present box-and-whisker plots for selected elements for which statistically significant differences were identified.

A thorough examination of the obtained results of the statistical analyses reveals that elements such as Ag, Al, Sn and Zr are indicative of samples containing aluminium–zirconium compounds as an antiperspirant substance (Table 2, Figure 7, Figure 8 and Figure 12). These are usually found in the stick form. However, in the case of deodorant products, where the active ingredients are natural products based on aluminium–potassium alum or those imitating natural products (e.g., calcium/magnesium/zinc hydroxyapatite), the most characteristic elements are Al, Ca, Cr, K, P, S, Sr, and Ti (Table 2, Figure 8, Figure 9, Figure 10 and Figure 11). By contrast, alcohol-based deodorants are distinguished by a significantly deficient elemental composition. A perusal of the ingredients listed on the labels of these products reveals that alcohol is among their primary constituents. Furthermore, the composition of these substances is notably organic, with a paucity of other, inorganic compounds. However, due to the limited representation of this group, which is solely represented by three samples in the study, it is not possible to make any generalisations about its prevalence.

### 2.3. Principal Component Analysis (PCA)

**Figure 13:**
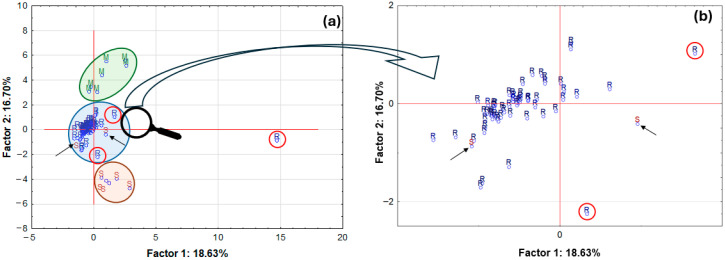
Projection of the cases on the factor-plane for the 72 samples of deodorants and antiperspirants divided by form: R—roll-on, M—mineral, S—stick (**a**) and enlarged chart area for the roll-on group R (**b**).

Performances of a principal component analysis (PCA) were undertaken to demonstrate the existence of any similarities between the groups which were analysed, and to successfully identify any potential outliers. It can be concluded from the PCA results that one of the tested samples, the roll-on deodorant sample (R) (Figure 13a), which contains the active ingredient calcium/magnesium/zinc hydroxyapatite, differs significantly from the remaining samples in the analysed set. This product is distinguished by its markedly divergent composition from the other samples, which is attributable to the presence of compounds exhibiting high mineral content. Furthermore, three distinct groups of samples can be distinguished on the graph: firstly, solid potassium aluminium alum samples (M); secondly, roll-on samples (R); and thirdly, stick products (S). It is noteworthy that in the expanded region pertaining to roll-on samples (Figure 13b), a closer alignment between the two samples designated as S (sticks) and the roll-on samples (R) is evident. The two samples under consideration are a deodorant and an antiperspirant. However, it should be noted that both samples contain alcohol. Consequently, a clear distinction emerges between these products and the other stick samples, as they predominantly utilise aluminium–zirconium compounds as their active ingredients.

**Figure 14:**
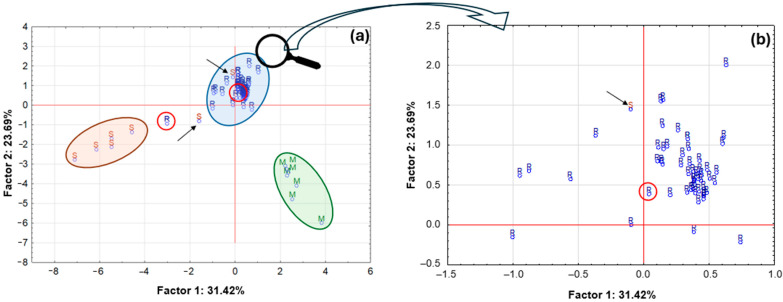
Projection of the cases on the factor-plane for the 71 samples of deodorants and antiperspirants divided by form: R—roll-on, M—mineral, S—stick (**a**) and enlarged chart area for the roll-on group R (**b**) after Ba, Co, Cu, Fe, Mn, Pb, Sb, Sr, Ti, Zn reduction.

Consequently, their composition aligns more closely with the category of samples available in roll-on form. It is also noteworthy that two roll-on samples deviate slightly from the group. The first sample (positioned at the uppermost point on the right-hand side of the circle) is an antiperspirant sample, which, according to the accompanying label, contains natural extracts, including pineapple extract. The second sample (positioned at the lowermost point, in closer proximity to the stick group) is also an antiperspirant sample, but it is one of a small number that contain an aluminium–zirconium compound as an antiperspirant ingredient. The total value of explained variance was only approximately 35%, so a partial data reduction was decided. Only data on element concentrations for which statistically significant differences were found were used for further analysis, and one outlier was removed. A graph of case projections onto the factorial plane after data reduction is presented in Figure 14.

A thorough examination of the resulting graph reveals that following the process of case reduction, three primary groups of samples have been identified (Figure 14a). The initial group comprised solid aluminium–potassium alums (M), from which a single specimen exhibited a marginal separation, characterised by a more substantial mineral composition in comparison to the remaining samples. In addition to the primary components, including Al, K, and S, the sample exhibited the presence of Ca, Cr, Fe, Mn, P, and Zn. The second group comprised five stick samples (S), while the third group consisted of roll-on samples. Following the reduction in the data, two samples, designated as ‘S’ and ‘R’, respectively, were found to be distinctly separate from their respective groups. The aluminium-based antiperspirant sample, as previously described, was utilised as the control group. Prior to the reduction in data, the aluminium-based antiperspirant sample exhibited a close proximity to the group of roll-on samples. The second sample was an antiperspirant in roll-on format, the composition of which exhibited a greater similarity to that of the stick sample, given its incorporation of an aluminium–zirconium compound. The previously documented roll-on antiperspirant sample, comprising pineapple extract, was incorporated into the roll-on sample group subsequent to data reduction. The second stick sample, however, is still included in the roll-on sample group and is a deodorant sample with alcohol as the active ingredient. The total value of variance explanation increased to approximately 55%.

### 2.4. SEM-EDS Results

The outlier sample, which contains calcium/magnesium/zinc hydroxyapatite, was subjected to a different testing technique, namely Scanning Electron Microscopy coupled with Energy-Dispersive X-ray Spectrometry (SEM-EDS). This technique also provides information on the sample’s elemental composition and surface morphology. In order to facilitate a meaningful comparison with other samples, the most representative samples from each group were selected and also tested using this technique. The application of an alternative research technique enabled the identification of additional elements that were not subject to analysis using the ICP-OES technique. Furthermore, this analysis yielded insights into the nature of the active substance employed in the cosmetic product.

**Figure 15:**
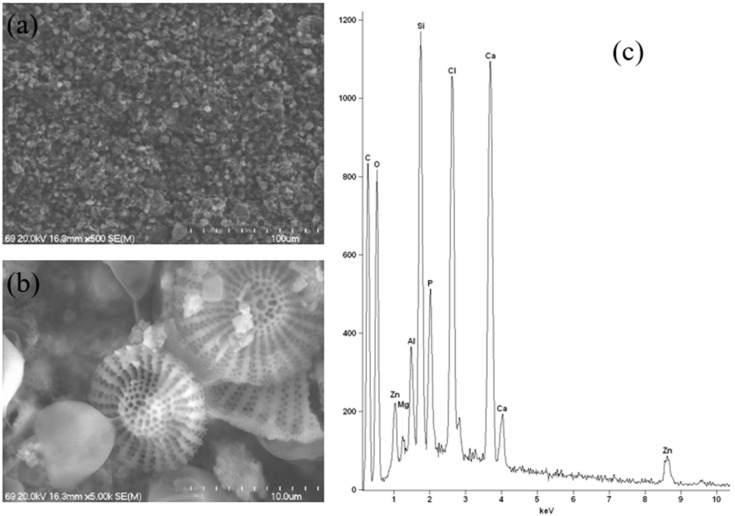
Scanning electron microscope images at 500× (**a**) and 5000× magnification (**b**) and energy-dispersive X-ray spectroscopy spectrum (**c**) of calcium/magnesium/zinc hydroxyapatite-containing deodorant.

A thorough analysis of the results obtained using SEM at various magnifications for a sample containing calcium/magnesium/zinc hydroxyapatite was performed. This analysis revealed the presence of various crystal sizes and characteristic structures resulting from the addition of diatomaceous earth. This finding was confirmed on the basis of microscopic images. Furthermore, the heterogeneity of the sample is evident. As previously referenced, the manufacturer also declares the presence of oryza sativa (rice) starch and clay minerals in the composition (Figure 15a,b). Natural hydroxyapatite is a complex of hydroxyl phosphates with calcium ions as their principal cation. Within the specific parameters of the hydroxyapatite mineral lattice, Ca^2+^ can be substituted by other ions, including Mg^2+^ and Zn^2+^. Concurrently, the phosphate and hydroxyl anions can be replaced by fluoride, chloride, or carbonate. This phenomenon occurs as a result of certain alterations to the crystalline lattice. Hydroxyapatite can be considered a mineral “sponge” in this context, given its capacity to adsorb ionic or polar substances. The species under consideration is contingent upon the composition of its environment [31]. This resulted in a new material that, when combined with other ingredients, could constitute a modern deodorant. A thorough analysis of the obtained EDS spectrum (Figure 15c) reveals that the elemental composition is consistent with that obtained using ICP-OES, with a double exception. The presence of significant quantities of chlorine and silicon in the sample is in accordance with the product label, which indicates that this deodorant contains, amongst other components, octenidine HCl and calcium chloride, which are responsible for the presence of chlorine in the sample, as well as mineral compounds which are responsible for the presence of silicon. It should be noted that the chlorine and silicon contents were not measured using ICP-OES.

**Figure 16:**
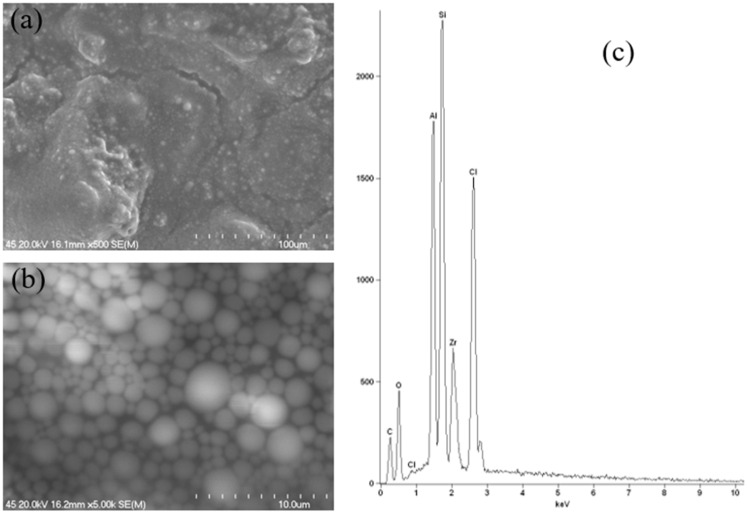
Scanning electron microscope images at 500× (**a**) and 5000× magnification (**b**) and energy-dispersive X-ray spectroscopy spectrum (**c**) of aluminium–zirconium-based antiperspirant.

As demonstrated in Figure 16, the results of the SEM-EDS analysis of a stick antiperspirant sample containing aluminium–zirconium tetrachlorohydre Gly as the active substance with a maximum content of zirconium are presented. The SEM images illustrate a globule-like structure of the sample, as the antiperspirant exhibited a gooey-gel consistency. Figure 16b, at a magnification of 5000×, serves to further illustrate this point, with the spherical structures exhibiting variability in size. Furthermore, this particular instance is characterised by a homogeneous nature. As demonstrated in Figure 16c, the EDS spectrum of the sample under investigation reveals the presence of aluminium (Al), zirconium (Zr), chlorine (Cl), oxygen (O) and silicon (Si). Consequently, the presence of both aluminium and zirconium in the antiperspirant sample that was subjected to testing was confirmed.

**Figure 17:**
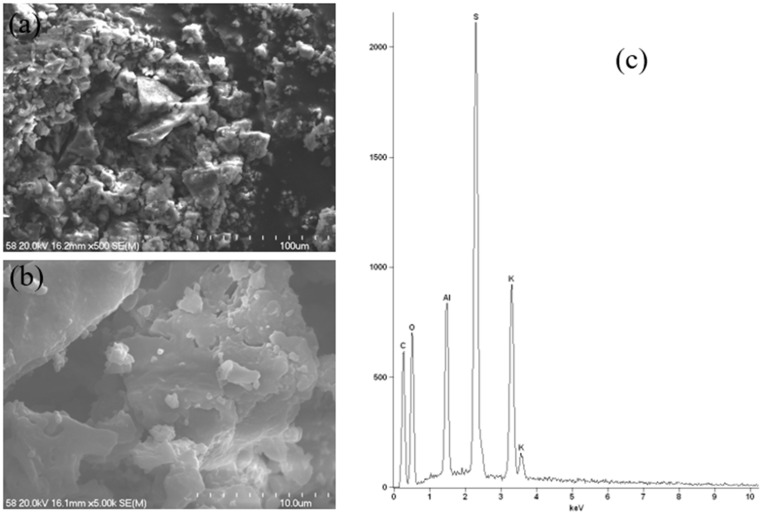
Scanning electron microscope images at 500× (**a**) and 5000× magnification (**b**) and energy-dispersive X-ray spectroscopy spectrum (**c**) of aluminium–potassium alum (deodorant).

As illustrated in Figure 17, the SEM-EDS results for a potassium–aluminium alum specimen with the highest aluminium content are presented. Alum is a crystalline substance, as can be seen in SEM images. The obtained EDS spectrum corresponds to the results obtained using the ICP-OES technique, where the main components were found to be S, K, and Al.

### 2.5. Study Limitations and Future Prospects

Even though that molecular composition was not part of this study, application of the SEM-EDS technique in addition to the other quantitative methods provided valuable complementary information. Combining scanning electron microscopy images with EDS spectra enabled more detailed characterization of the samples and supported more complete identification of their elemental composition. This multidimensional approach improves the interpretability of the results and paves the way for the integration of morphological and compositional data in future analyses.

Another limitation of the present study is that the sample preparation procedure did not guarantee complete decomposition of the antiperspirant matrices. However, the analytical approach focused on the extractable (leachable) elemental content, which more accurately reflects the exposure levels under “consumer use conditions”. Thus, this study aligns with the practical aspects, especially in the context of potential dermal absorption. In future research, complete digestion protocols could be applied to assess total elemental content, allowing for a more comprehensive comparison between extractable and total concentrations. Additionally, expanding the study to include a broader range of products (types and brands), as well as investigating the speciation of key elements, would provide deeper insight into potential health risks and regulatory implications.

Although the present study did not include a toxicological risk assessment, future research could build on the current findings by incorporating exposure-based simulations using bioaccessibility and bioavailability data. This approach would provide a more comprehensive and realistic estimation of the potential health risks associated with antiperspirant use. Particular attention should be given to elements such as aluminum (Al) and zirconium (Zr), which are commonly used in such formulations but whose long-term biological effects, including systemic absorption and cellular interactions, remain insufficiently characterized.

Moreover, given the dynamic nature of the cosmetics market, continuous monitoring of antiperspirant products remains essential. Formulations and ingredients are subject to frequent changes driven by consumer trends, technological innovation, and evolving safety standards. In particular, the growing consumer preference for so-called “natural” cosmetics introduces new raw materials and formulations whose elemental composition and safety profiles may differ significantly from conventional products. Therefore, future studies should be conducted in alignment with current regulatory frameworks, which themselves may be updated over time to reflect new scientific evidence and public health priorities.

## 3. Materials and Methods

### 3.1. Samples

The study examined the antiperspirant and deodorant samples procured from various commercial outlets, including brick-and-mortar stores, beauty supply stores, and online retailers, all operating within the Polish market. The deodorant and antiperspirant samples tested were supplied by a range of well-known global cosmetic brands and small local companies. A variety of brands were sampled, with some samples being sourced from the same manufacturer. Samples were collected and tested between 2022 and 2025. The majority of studied deodorants and antiperspirants that have been examined are available within the European market. The study did not involve the analysis of spray samples. The resultant dataset comprised a total of 72 samples, categorised according to type (deodorant, antiperspirant), formulation (roll-on—R, stick—S, mineral—M) and the type of active ingredient contained in the deodorant or antiperspirant (antiperspirants containing various forms of aluminium, antiperspirants containing both aluminium and zirconium, deodorants containing minerals such as aluminium–potassium alum, and other compounds as alcohol). The division of the samples was achieved through analysis of the product composition in accordance with the information provided on their respective labels. A comprehensive analysis of the samples and the abbreviations employed in this study are provided in Table 3.

The majority of the samples analysed were antiperspirants (n = 57), as the primary objective of the study was to verify the elemental composition, particularly the content of Al and Zr, in antiperspirants. Given the ubiquity of deodorants, they were included in the study, albeit in minimal quantities, for the purpose of comparison (n = 15). Samples labelled “antiperspirant deodorant” were included in the antiperspirant group due to the aluminium hydrochloride content declared on their labels.

In addition, the majority of the examined samples were found to be in roll-on form, which is the most common form of both deodorants and antiperspirants (n = 58). The samples included in the study were typical antiperspirants containing aluminium compounds, aluminium–zirconium compounds, and deodorants. The second most prevalent form was sticks, which primarily comprised aluminium–zirconium compounds as the active ingredient (n = 7). The third group consisted of aluminium–potassium alum, which are classified as deodorants (n = 7).

The tested samples were also divided according to the content of substances with antiperspirant properties (antiperspirants) and deodorant properties (deodorants). Therefore, the samples were divided into:

(a) Those containing aluminium compounds: aluminium chlorohydrate, aluminium chloride, aluminium sesquichlorohydrate, and aluminium lactate (n = 49);
(b) Aluminium–zirconium compounds containing aluminium zirconium pentachlorhydrate, aluminium zirconium tetrachlorohydrex GLY, and aluminium zirconium octachlorohydrex GLY (n = 8);
(c) Compounds based on natural mineral deodorants such as aluminium–potassium/aluminium–magnesium alum, calcium/magnesium/zinc hydroxyapatite, oryza sativa (rice) starch, diatomaceous earth, clay minerals (n = 12);
(d) Alcohols—primarily alcohol denat. (although information on the type of alcohol used is not always provided) (n = 3).

The testing process also included the analysis of substances known as sweat blockers, which have been found to contain aluminium compounds and alcohol denatured. However, they were classified as products containing aluminium compounds as the active ingredient. In the majority of cases, the samples consist of a mixture of various synthetic organic compounds, some of which also contain plant extracts (allantoin, aloe barbadensis leaf extract, helianthus annuus seed oil, mentha arvensis leaf oil), which significantly modify the elemental composition, especially with respect to macroelements.

### 3.2. Sample Preparation

All aqueous solutions were prepared with analytical-grade reagents and ultrapure water, resistivity higher 18.2 MΩcm (Milli-Q^®^, Millipore, Bedford, MA, USA). Prior to analysis, deodorants and antiperspirants were unpacked. The samples were weighed into test tubes using an analytical balance, with a range of between 0.15 and 0.20 g. Subsequently, 2 mL of 69% HNO_3_ (Suprapur, Merck, Darmstadt, Germany) and 1 mL of 36.5–38.0% HCl (J.T. Baker, Avantor, Radnor, PA, USA) were added. In instances where samples contained notable quantities of alcohol, acids were introduced in 0.2 mL increments due to the exothermic reaction, which was found to be highly rapid. The mineralisation of the samples was accomplished through the utilisation of the microwave-assisted closed-loop mineralisation system (UltraWave system, Milestone, Via Fatebenefratelli, Italy). The digestion process of the material under investigation was carried out in accordance with the selected organic sample digestion program (with an extended time for the second mineralization stage). The process comprised two stages, the parameters of which are presented in Table 4. The selected programme was also applied to the tested CRMs and blank material.

Following mineralisation, the resulting solutions were quantitatively transferred to volumetric flasks, with the addition of an internal standard (In, Sigma-Aldrich, Buchs, Switzerland) and made up to a volume of 25 mL. The solutions were prepared in appropriate replicates. In certain samples, it was not possible to obtain clear solutions after the mineralisation process (fine suspension was present), in which case the samples were subjected to centrifugation prior to measurement (OHAUS centrifuge, Wehingen Germany, 6000 rpm, centrifugation time 10 min).

In the absence of a commercially available certified reference material with this matrix, it was decided to test a material containing high concentrations of aluminium and zirconium, as well as a number of other trace elements. Reference materials containing zirconium are much less common due to the low abundance of this element in nature. The study made use of MODAS-2 Bottom Sediment (Laboratory of Nuclear Analytical Methods, Warsaw, Poland), a material that has been certified as such. This material contains 3.77 ± 0.39% Al (an element with a certified concentration value) and 434 mg/kg Zr (an element with an informative concentration value).

### 3.3. Instrumentation

#### 3.3.1. ICP-OES

The present study was conducted using a Thermo Scientific™ iCAP™ 7400 ICP-OES analyser, which was equipped with a standard aqueous sample introduction kit. The instrument parameters utilised for the analysis are delineated in Table 5. The Thermo Scientific iCAP 7000 Series ICP-OES analyser (Waltham, MA, USA) incorporates high-resolution echelle optics and an enhanced fourth-generation charge injection detector (CID). Advancements in CID technology have enabled this detector to achieve enhanced sensitivity and reduced noise levels when compared to its predecessors. Collectively, these enhancements render the instrument optimal for precise and accurate measurements of samples containing both macro and trace elements.

In order to measure the indicated elements, it was necessary to prepare calibration curves based on a 100 mg/L CPAchem standard solution (ICP multielement standard, Stara Zagora, Bulgaria) and several single-element standards: the concentrations of S and P were set at 1000 mg/L (ICP grade, Analytika, Prague, Czech Republic) and 1000 mg/L (ICP grade, Radian International LLC, Austin, TX, USA), respectively. The standard preparation method employed was the dilution technique.

The linear regression coefficient for each analyte ranged from 0.998 to 1.000. The sensitivity of the developed method was evaluated in terms of the limit of quantification (LOQ) (Table 6). The RSD, expressed as a percentage, ranged from 0.01 to 5.00%, even for elements measured at very low concentrations. The accuracy of the procedure was verified by analysis of certified reference materials. The obtained recoveries ranged from 88.6 to 114.7%.

The measurements employed an internal standard (In), which, when added in a known quantity, generates an analytical signal similar in nature to the substances being analysed in the samples. This process enables the rectification of the deleterious impact of uncontrolled factors on measurement outcomes, thereby ensuring enhanced accuracy and reliability.

#### 3.3.2. SEM-EDS

For the purpose of SEM-EDS analysis, the selected samples were initially positioned on plastic dishes before being subjected to drying in a laboratory incubator until a moisture-free state was achieved (BINDER, Tuttlingen, Germany).

The morphology and elemental composition of the samples were examined using a field emission scanning electron microscope (SEM, Hitachi S-4700, Tokyo, Japan) equipped with an energy-dispersive X-ray detector (EDS, Thermo Scientific UltraDry, Madison, WI, USA).

SEM imaging was performed at an accelerating voltage of 20 kV with magnifications ranging with 500× and 5000×. The morphology of the surface was observed using secondary electron (SE) imaging.

Elemental composition and distribution mapping were analyzed using the EDS detector under the same conditions.

### 3.4. Data Analysis

The statistical and multivariate analysis was conducted using Statistica 12.5 (New York, NY, USA) software.

In order to assess the consistency of the data distribution for all samples analysed at the assumed significance level (*p* = 0.05) with the normal distribution, the Shapiro–Wilk and Kolmogorov–Smirnov tests were used. Consequently, it was concluded that the data did not meet the assumption of normal distribution. Further analysis was conducted utilising statistical methodologies appropriate for data that did not demonstrate a normal distribution (the non-parametric Kruskal–Wallis test). The objective of the present study was to utilise the aforementioned test to evaluate the significance of discrepancies in the determined levels of elements between individual groups, depending on the parameters considered, such as product type, form, and the type of active ingredient contained.

## 4. Conclusions

The elemental composition of the tested deodorant and antiperspirant samples was found to be highly diverse. The division of all samples into groups facilitated a comprehensive characterisation of the studied groups, particularly the roll-on (R) samples. However, due to the limited representation of certain samples, such as alcohol-based deodorants (a), the characterisation of this group was incomplete. It is possible to create a series of content concentrations (descending) based on the median concentrations of the analysed elements. The following content series can be created, listed in descending order:

(a) For samples in the form of roll-ons (R):

Al >> K > Ca ≈ S ≈ Fe > Zn ≈ P > Ti > Zr > Sb ≈ Mn ≈ Ni > Cr ≈ Sr > Ba ≈ Cu ≈ Sn > Mg.

(b) For samples in the form of solid aluminium–potassium alums (M):

S >> K >> Al >> Ca > Fe > P > Ti > Cr ≈ Zr ≈ Zn > Sr > Ni > Cu.

(c) For samples in the form of sticks (S):

Al > Zr >> Mg > Ag > Ca > K > S > Fe > Sn > Ti > Zn > Mn > Pb > Cu > Sr.

(d) For deodorants (D):

S >> K > Al > Ca > Fe ≈ P > Ti > Zr > Zn > Cr ≈ Sr > Ni > Sb ≈ Cu > Mn.

(e) For antiperspirants (A):

Al >> K > Ca ≈ S ≈ Fe > Zn > P >Ti > Zr > Mn > Sb > Ni > Cr > Sr > Cu ≈ Sn > Ba ≈ Ag > Mg.

Consequently, it can be deduced that aluminium constitutes a pivotal component in both antiperspirants (in various forms) and deodorants derived from natural aluminium–potassium alum. The concentrations of aluminium obtained in this study are not in excess of the recommended aluminium concentration in non-spray products as stipulated by the Scientific Committee on Consumer Safety. In contrast, aluminium and zirconium are main constituents of antiperspirants in the stick form.

When considering elements such as Ba, Sb, Sn, and Ti, which may be present in cosmetic products under certain conditions (most often as chemical compounds imparting colour), it can be concluded that the concentrations of these elements in the tested samples are not high. However, isolated elevated values do occur. A parallel conclusion can be drawn regarding the presence of antimony in cosmetic products, a constituent that is proscribed in such formulations. However, the situation is different for heavy metals such as Cd and Pb, whose use in cosmetics is prohibited. Cadmium concentrations above the LOQ were not quantified in any of the samples. Nevertheless, lead was quantified in 37 of the 72 samples, with the highest value obtained for natural potassium aluminium alum in stone, namely 15.90 mg/L. In light of the findings, it can be concluded that there is a necessity for more stringent supervision of cosmetic products.

The measurements performed enabled the identification of one outlier sample, the composition of which differed significantly from all other samples. Furthermore, it was demonstrated that the simultaneous use of multiple research techniques allows for the supplementation of data obtained with a single technique. Despite the fact that SEM-EDS is a non-obvious technique for examining this type of sample, difficult analysis can yield interesting results that provide much more information about the sample being examined. In conclusion, advances in technology and various fields of science have led to the development of new deodorant preparations on a continuous basis. Nonetheless, the fundamental ingredients of antiperspirants remain hydrated aluminium salts or aluminium–zirconium complexes.

## Figures and Tables

**Table 1:** The mean concentration and median value of each element content in deodorants and antiperspirants divided by form: R—roll-on, M—mineral, S—stick [mg/kg].

Element	Form	n	Mean	Median	Element	Form	n	Mean	Median
Ag	R	58	4.674	<LOQ	Ni	R	58	0.503	0.229
	M	7	0.035	<LOQ		M	7	0.130	0.123
	S	7	131.7	188.4		S	7	<LOQ	<LOQ
Al	R	58	24,340	28,200	P	R	58	62.55	2.537
	M	7	39,680	36,280		M	7	8.726	7.444
	S	7	26,000	26,840		S	7	0.062	<LOQ
Ca	R	58	127.1	15.11	S	R	58	448.4	14.32
	M	7	36.97	27.51		M	7	102,300	104,680
	S	7	85.10	71.31		S	7	56.19	35.03
Cr	R	58	0.185	0.135	Sn	R	58	0.402	0.058
	M	7	1.810	1.230		M	7	0.017	<LOQ
	S	7	<LOQ	<LOQ		S	7	7.707	6.424
K	R	58	307.5	46.26	Zr	R	58	511.5	0.687
	M	7	61,340	56,220		M	7	2.854	1.060
	S	7	53.05	40.60		S	7	17,580	21,420
Mg	R	58	8.353	0.022					
	M	7	0.468	<LOQ					
	S	7	389.1	426.7					

**Table 2:** The mean concentration and median value of each element content in deodorants and antiperspirants divided by the type of primary deodorant or antiperspirant ingredient they contained: aluminium-based products (Al), aluminium–zirconium-based products (AlZr), natural compound-based products (N), alcohol-based products (a) [mg/L].

Element	Active Substance	n	Mean	Median	Element	Active Substance	n	Mean	Median
Ag	Al	49	1.577	<LOQ	Ni	Al	49	0.584	0.240
	AlZr	8	139.4	166.2		AlZr	8	<LOQ	<LOQ
	N	12	0.030	<LOQ		N	12	0.112	0.110
	a	3	<LOQ	<LOQ		a	3	0.037	<LOQ
Al	Al	49	28,210	29,670	P	Al	49	4.224	2.249
	AlZr	8	24,800	25,070		AlZr	8	<LOQ	<LOQ
	N	12	24,190	34,980		N	12	288.8	10.44
	a	3	33.23	38.84		a	3	5.534	4.050
Ca	Al	49	29.39	12.73	S	Al	49	28.76	13.22
	AlZr	8	68.63	52.77		AlZr	8	98.98	36.98
	N	12	515.5	31.65		N	12	61,690	94,130
	a	3	18.06	20.79		a	3	21.71	31.15
Cr	Al	49	0.209	0.182	Sn	Al	49	0.240	0.059
	AlZr	8	<LOQ	<LOQ		AlZr	8	7.869	6.346
	N	12	1.093	0.453		N	12	0.044	<LOQ
	a	3	<LOQ	<LOQ		a	3	0.711	0.764
Cu	Al	49	1.061	0.066	Sr	Al	49	0.233	0.123
	AlZr	8	0.539	0.540		AlZr	8	0.358	0.289
	N	12	0.108	0.057		N	12	1.261	0.326
	a	3	0.055	<LOQ		a	3	<LOQ	<LOQ
K	Al	49	60.79	45.16	Ti	Al	49	2.126	1.665
	AlZr	8	52.88	41.06		AlZr	8	1.799	1.368
	N	12	36,700	53,210		N	12	7.419	7.550
	a	3	63.33	62.48		a	3	0.148	0.205
Mn	Al	49	0.414	0.395	Zr	Al	49	187.2	0.527
	AlZr	8	0.578	0.592		AlZr	8	17,940	21,210
	N	12	0.534	0.064		N	12	2.532	1.193
	a	3	<LOQ	<LOQ		a	3	1.136	1.288

**Table 3:** Characteristics of the tested deodorants and antiperspirants and abbreviations used in the article.

n = 72		
Type	Form	Active Substance
Deodorant (D) n = 15	Roll-on (R) n = 58	Aluminium-based products (Al) n = 49
Antiperspirant (A) n = 57	Stick (S) n = 7	Aluminium–zirconium-based products (AlZr) n = 8
	Mineral (M) n = 7	Natural compound-based products (N) n = 12
		Alcohol-based products (a) n = 3

**Table 4:** Microwave digestion process conditions.

Stage	Time [min]	E [W]	T_1_ [°C]	T_2_ [°C]	P [bar]
1	15	1500	220	70	120
2	20	1500	220	70	120

**Table 5:** Instrumental conditions and operating parameters for ICP-OES.

Instrument Parameter	Operating Conditions
Generator power [W]	1150
Carrier gas	Argon
Plasma gas flow rate [L/min]	12
Auxiliary gas flow rate [L/min]	0.5
Nebulizer gas flow rate [L/min]	0.5
Nebulizer	Concentric quartz
Spray chamber	Cyclonic quartz
Torch	Quartz
Replicates per sample	3
Nebulizer gas pressure [kPa]	250

**Table 6:** LOQs values for the analytical procedure proposed for deodorant and antiperspirant analysis using ICP-OES.

Analyte	LOQ [mg/L]	Analyte	LOQ [mg/L]
Ag 328.068 ^a^	0.005	Ni 231.604 ^a^	0.010
Al 396.152 ^a^	0.031	P 177.495 ^a^	0.044
Ba 455.403 ^r^	0.020	Pb 182.205 ^a^	0.011
Ca 393.366 ^r^	0.042	S 180.731 ^a^	0.088
Cd 214.438 ^a^	0.005	Sb 206.833 ^a^	0.017
Co 228.616 ^a^	0.005	Sn 189.989 ^a^	0.028
Cr 267.716 ^a^	0.031	Sr 421.552 ^r^	0.008
Cu 224.700 ^a^	0.007	Ti 334.941 ^a^	0.0010
Fe 259.940 ^a^	0.090	Zn 213.856 ^a^	0.037
K 766.490 ^r^	0.99	Zr 339.198 ^a^	0.031
Mg 279.553 ^r^	0.047	In * 230.606 ^a^	0.016
Mn 257.610 ^a^	0.004		

^a^—axial view; ^r^—radial view; *—internal standard.

## Data Availability

The data presented in this study are available on request from the corresponding author.

## Abbreviations

The following abbreviations are used in this manuscript:

a	alcohol-based products
A	antiperspirant
ACH	aluminium hydrochloride
Al	aluminium-based products
AlZr	aluminium–zirconium-based products
CID	charge injection detector
D	deodorant
FAAS	Flame Atomic Absorption Spectrometry
FIA	Flow Injection Analysis
HA	hydroxyapatite
ICP-OES	Inductively Coupled Plasma-Optical Emission Spectrometry
M	mineral
N	natural-based products
LOQ	limit of quantification
PCA	Principal Component Analysis
R	roll-on
S	stick
SCCS	Scientific Committee on Consumer Safety
SEM-EDS	Scanning Electron Microscopy/Energy-Dispersive X-ray Spectrometry
Zinc PCA	Zinc Pyrrolidone Carboxylic Acid

## References

[1] Lader K. (1999). Antiperspirants and Deodorants.

[2] de Oliveira E.C.V., Salvador D.S., Holsback V., Shultz J.D., Michniak-Kohn B.B., Leonardi G.R. (2021). Deodorants and antiperspirants: Identification of newstrategies and perspectives to prevent and control malodorand sweat of the body. Int. J. Dermatol..

[3] Teerasumran P., Velliou E., Bai S., Cai Q. (2023). Deodorants and antiperspirants: New trends in their active agents and testing methods. Int. J. Cosmet. Sci..

[4] Klepak P., Walkey J., Butler H. (2000). Antiperspirants and deodorants. Poucher’s Perfumes, Cosmetics and Soaps.

[5] Bhatt H.B., Patel N.B. (2021). Natural Deodorants: A way towards sustainable cosmetics. Int. J. Pharm. Sci. Health Care.

[6] Kalinowska-Lis U. (2025). Overview of Active Ingredients Used in Deodorants and Antiperspirants Available on EU Market. Appl. Sci..

[7] Urban J., Fergus D.J., Savage A.M., Ehlers M., Menninger H.L., Dunn R.R., Horvath J.E. (2016). The effect of habitual and experimental antiperspirant and deodorant product use on the armpit microbiome. PeerJ.

[8] Alzomor A.K., Moharram A.S., Al Absi N.M. (2014). Formulation and evaluation of potash alum as deodorant lotion and after shaving astringent as cream and gel. Int. Curr. Pharm. J..

[9] Roslan F.K., Taib C., Azhar A.A., Ab Razak S.N. (2025). Zinc Oxide as an Active Ingredient in Sensitive Skin Deodorant. MARI.

[10] European Commission (2009). Regulation (EC) No 1223/2009 of the European Parliament and of the Council of 30 November 2009 on Cosmetic Products.

[11] Scientific Committee on Consumer Safety (2020). SCCS Opinion on the Safety of Aluminium in Cosmetic Products, Submission II, SCCS/1613/19.

[12] Exley C. (2016). The toxicity of aluminium in humans. Morphologie.

[13] McGrath K.G. (2009). Apocrine sweat gland obstruction by antiperspirants allowing transdermal absorption of cutaneous generated hormones and pheromones as a link to the observed incidence rates of breast and prostate cancer in the 20th century. Med. Hypotheses.

[14] Pineau A., Fauconneau B., Sappino A.-P., Deloncle R., Guillard O. (2014). If exposure to aluminium in antiperspirants presents health risks, its content should be reduced. J. Trace Elem. Med. Biol..

[15] Moussaron A., Alexandre J., Chenard M.-P., Mathelin C., Reix N. (2023). Correlation between daily life aluminium exposure and breast cancer risk: A systematic review. J. Trace Elem. Med. Biol..

[16] Darbre P.D., Mannello F., Exley C. (2013). Aluminium and breast cancer: Sources of exposure, tissue measurements and mechanisms of toxicological actions on breast biology. J. Inorg. Biochem..

[17] Hangan T., Bjorklund G., Chirila S. (2025). Exploring the Potential Link between Aluminum-Containing Deodorants/Antiperspirants and Breast Cancer: A Comprehensive Review. Curr. Med. Chem..

[18] Klotz K., Weistenhöfer W., Neff F., Hartwig A., van Thriel C., Drexler H. (2017). The health effects of aluminum exposure. Dtsch. Arztebl. Int..

[19] Amadi L.O. (2020). A Review of Antimicrobial Properties of Alum and Sundry Applications. Acta Sci. Microbiol..

[20] Correia da Costa W.K.O., Santos da Silva C., Dagnone Figueiredo J.F., Araujo Nóbrega J., Silveira Paim A.P. (2018). Direct analysis of deodorants for determination of metals by inductively coupled plasma optical emission spectrometry. J. Pharm. Biomed. Anal..

[21] Mayildurai R., Ramasubbu A., Velmani N. (2015). ICP-OES investigations of heavy metal contents in cosmetic products. J. Pharm. Res..

[22] Khadim R.y., Alwan N.k., Noori Z.A., Thahab N.M. (2023). Determination of Lead and Aluminum in Selected Antiperspirant using Atomic Absorption Spectrometry in Baghdad City/Iraq. Cent. Asian J. Med. Nat. Sci..

[23] Kasim L.S., Ogunnubi B., Dawodu O.J., Olaitan J.O., Ayodele O. (2013). Quantitative assessment of metals in some antiperspirant formulations marketed in Nigeria. JPSI.

[24] Teixeira G.G., Virgilio A., Santos P.M. (2023). Direct Determination of the Aluminum Content in Antiperspirants Using Digital Image Colorimetry. Chem. Sel..

[25] Polli Silvestre A.L., Milani M.I., Rossini E.L., Pezza L., Redigolo Pezza H. (2018). A paper platform for colorimetric determination of aluminum hydrochloride in antiperspirant samples. Spectrochim. Acta A Mol. Biomol. Spectrosc..

[26] Chiu M.-H., Kumar A.S., Sornambikai S., Zen J.-M., Shih Y. (2010). Flow Injection Analysis of Aluminum Chlorohydrate in Antiperspirant Deodorants Using a Built-in Three-in-one Screen-Printed Silver Electrode. Electroanalysis.

[27] Jasiorski M., Leszkiewicz A., Brzeziński S., Bugla-Płoskońska G., Malinowska G., Borak B., Karbownik I., Baszczuk A., Stręk W., Doroszkiewicz W. (2009). Textile with silver silica spheres: Its antimicrobial activity against Escherichia coli and Staphylococcus aureus. J. Sol-Gel Sci. Technol..

[28] Li W.-R., Xie X.-B., Shi Q.-S., Zeng H.-Y., Ou-Yang Y.-S., Chen Y.-B. (2010). Antibacterial activity and mechanism of silver nanoparticles on Escherichia coli. Appl. Microbiol. Biotechnol..

[29] Szczepańska E., Bielicka-Giełdoń A., Niska K., Strankowska J., Żebrowska J., Inkielewicz-Stępniak I., Łubkowska B., Swebocki T., Skowron P., Grobelna B. (2020). Synthesis of silver nanoparticles in context of their cytotoxicity, antibacterial activities, skin penetration and application in skincare products. Supramol. Chem..

[30] Rastrelli G., Rastrelli F., Tosti G., Deola M., Rigano L. (2020). The biomimetic hydroxyapatite was found to effectively absorb sweat without irritation and while improving skin wellness and homeostasis. Cosmet. Toilet..

[31] Younes M., Aquilina G., Castle L., Engel K.-H., Fowler P., Frutos Fernandez M.J., Fürst P., Gürtler R., Gundert-Remy U., EFSA Panel on Food Additives and Flavourings (FAF) (2019). Opinion on the follow-up of the re-evaluation of sorbic acid(E200) and potassium sorbate (E202) as food additives. EFSA J..

[32] Nedjimi B. (2025). New Insights into Trace Element Accumulation in Kernels of Two Algerian Populations of Argan (*Argania spinosa* (L.) Skeels): An Endangered Endemic Tree. Biol. Trace Elem. Res..

